# A case report of erythroderma in a patient with borderline leprosy on reversal reaction: a result of the exacerbated reaction?

**DOI:** 10.1186/s12895-017-0068-3

**Published:** 2017-12-20

**Authors:** Denis Miyashiro, Ana Paula Vieira, Maria Angela Bianconcini Trindade, João Avancini, José Antonio Sanches, Gil Benard

**Affiliations:** 1 Department of Dermatology, Hospital das Clínicas, University of São Paulo Medical School, São Paulo, Brazil; 2 Clinical and Experimental Allergy and Immunology Laboratory LIM-56, University of São Paulo Medical School, São Paulo, Brazil

**Keywords:** Erythroderma, Leprosy, Reversal reaction, Regulatory T-cells

## Abstract

**Background:**

Erythroderma is characterized by erythema and scaling affecting more than 90% of the body surface area. Inflammatory, neoplastic and, more rarely, infectious diseases may culminate with erythroderma. Diagnosis of the underlying disorder is therefore crucial to institute the appropriate therapy. Leprosy is a chronic infectious disease that is endemic in Brazil. Here we present an unusual case of leprosy and reversal reaction causing erythroderma, and we discuss the underlying immunological mechanisms which could contribute to the generalized skin inflammation.

**Case presentation:**

We report a case of a patient with reversal reaction (RR) in borderline borderline leprosy presenting with erythroderma and neural disabilities. Histopathology of the skin showed regular acanthosis and spongiosis in the epidermis and, in the dermis, compact epithelioid granulomas as well as grouped and isolated bacilli. This duality probably reflects the transition from an anergic/multibacillary state to a state of more effective immunity and bacillary control, typical of RR. Leprosy was successfully treated with WHO’s multidrug therapy, plus prednisone for controlling the RR; the erythroderma resolved in parallel with this treatment. Immunologic studies showed in situ predominance of IFNγ + over IL-4+ lymphocytes and of IL-17+ over Foxp3+ lymphocytes, suggesting an exacerbated Th-1/Th-17 immunoreactivity and poor Th-2 and regulatory T-cell responses. Circulating Tregs were also diminished. We hypothesize that the flare-up of anti-mycobacteria immunoreactivity that underlies RR may have triggered the intense inflammatory skin lesions that culminated with erythroderma.

**Conclusions:**

This case report highlights the importance of thorough clinical examination of erythrodermic patients in search for its etiology and suggests that an intense and probably uncontrolled leprosy RR can culminate in the development of erythroderma.

**Electronic supplementary material:**

The online version of this article (10.1186/s12895-017-0068-3) contains supplementary material, which is available to authorized users.

## Background

Erythroderma is the maximal stage of skin inflammation, with erythema and scaling affecting more than 90% of the body surface area, and is considered a dermatologic emergency. Several diseases may culminate with erythroderma: exacerbation of preexisting dermatoses (psoriasis, atopic dermatitis, eczema), drug reactions, and cutaneous lymphomas. Erythroderma is rarely caused by infections (scabies, dermatophytosis) [1]. With the possible exception of two cases of leprosy patients presenting the erythroderma-related “deck-chair” sign, it has not been associated with leprosy [2, 3].

Leprosy is still endemic in several countries, including Brazil. It can cause severe skin alterations, neural disability, and, consequently, social and functional stigmas. Skin lesions are polymorphic, ranging from a single hypochromic hypoesthetic macule to diffuse skin infiltration [4]. This polymorphism may delay diagnosis, leading to progression of disabilities and increased risk of transmission.

Here we present an unusual case of leprosy and reversal reaction causing erythroderma, and we discuss the underlying immunological mechanisms which could contribute to the generalized skin inflammation.

## Case presentation

**Fig. 1:**
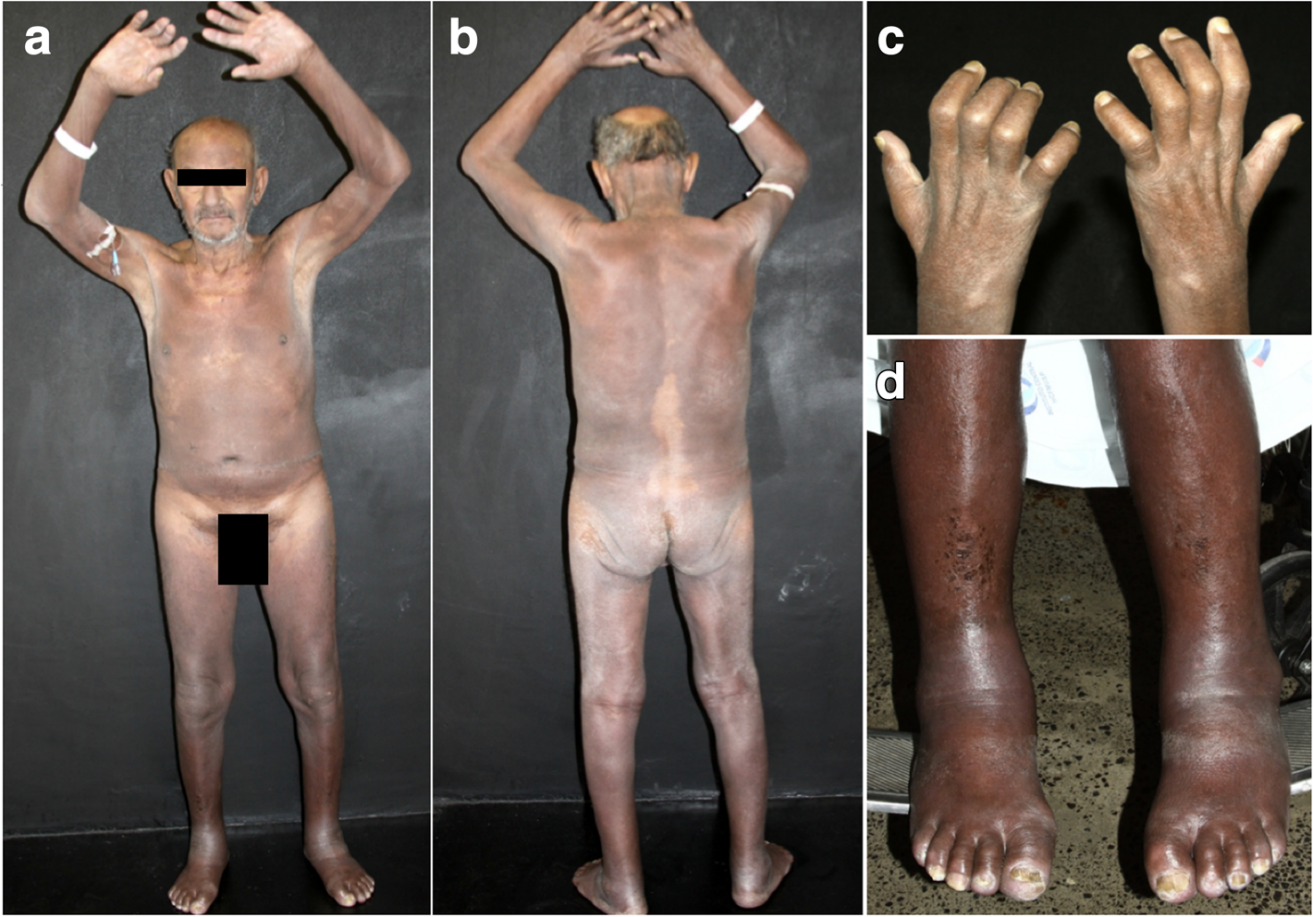
Clinical Findings. **a** Erythroderma: diffuse erythema and infiltration of the skin. Axillary and inguinal areas are spared. **b** Erythroderma: diffuse erythema and infiltration of the skin. Lumbar area is spared. **c** Amyotrophy of interosseous muscles of the hands and oedema of the fingers. **d** Oedema of lower limbs

A 63-year-old man presented with a two-month history of erythroderma, with diffuse infiltration of the skin. Axillary and inguinal areas were spared (Fig. 1a, b). Neurological examination disclosed amyotrophy of interosseous muscles of the hands, ulnar claw, paresthesia of hands and feet, and thickening of ulnar and fibular nerves (Fig. 1c). There was pronounced oedema of hands and feet (Fig. 1d). He had no history of skin diseases or exposure to new medications or allergens. The main initial hypothesis was cutaneous lymphoma due to the diffuse infiltration of the skin; however, the neurologic signs and symptoms raised the suspicion of leprosy.

**Fig. 2:**
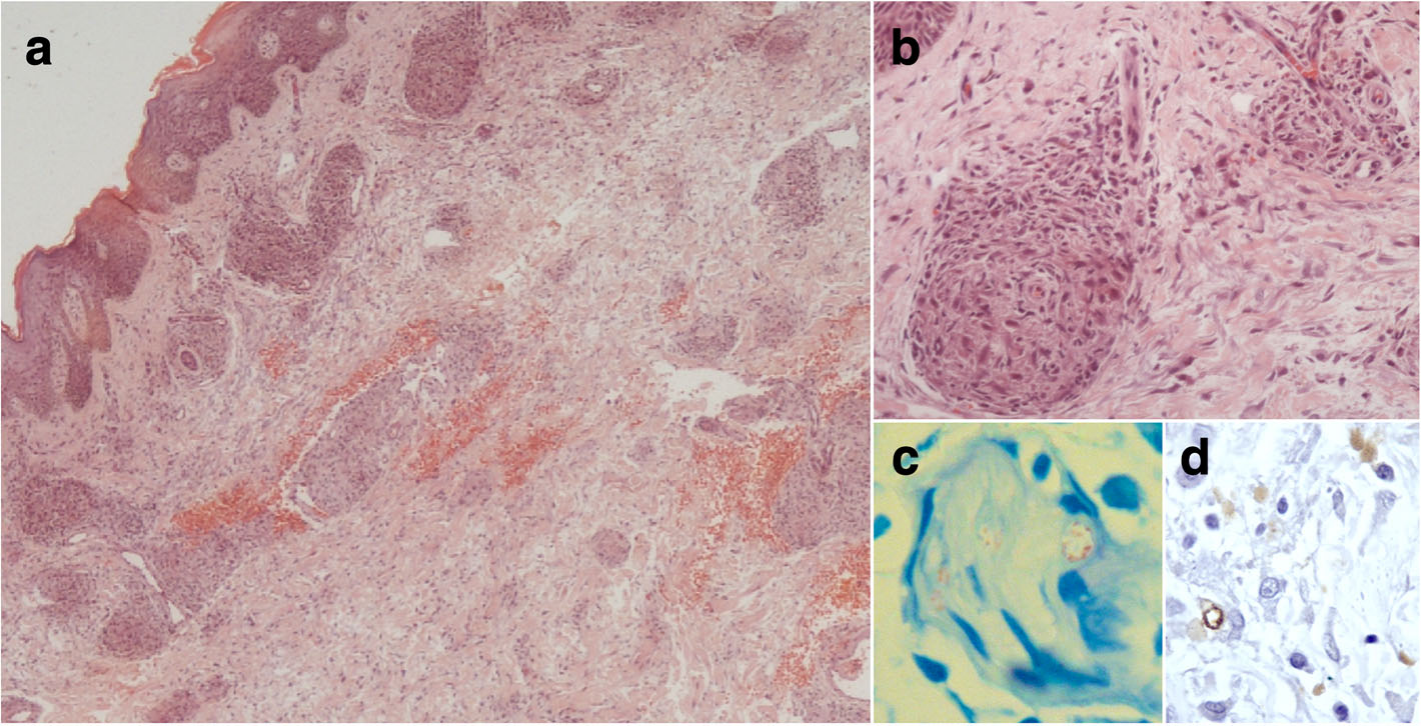
Histopathological findings. **a** Epidermis with regular acanthosis and spongiosis. Dermis with lympho-histiocytic infiltrate in perivascular and periadnexial patterns, congestion and enlargement of vessels, and extravasation of erythrocytes (haematoxylin-eosin, original magnification ×40). **b** Granulomas with epithelioid histiocytes, associated with discrete oedema and infiltration by lymphocytes (haematoxylin-eosin, original magnification ×200). **c**. Fite-Faraco staining showing a nerve circumscribed by lympho-histiocytic infiltrate with grouped acid-fast bacilli (original magnification ×1000). **d** Positive anti-BCG staining (original magnification ×400)

Skin biopsies revealed epidermis with regular acanthosis, spongiosis and dermis with chronic epithelioid granulomatous infiltration in perivascular, periadnexial and perineural patterns (Fig. 2a, b). Fite-Faraco staining showed isolated and grouped acid-fast bacilli (Fig. 2c). Immunohistochemistry with anti-BCG was positive within nerves and inflammatory cells (Fig. 2d). Diagnosis of reversal reaction (RR) in borderline borderline leprosy was made and the erythroderma was linked to the reaction. The patient was born in the countryside of Minas Gerais state, a highly endemic area for leprosy, but had been living in São Paulo city, that has low endemicity, for the last 23 years. There was no family history of leprosy. The patient was HIV negative.

Multidrug therapy with dapsone, clofazimine, and rifampicin (MDT) based on WHO recommendation was initiated. RR-associated neuritis was treated with prednisone 40 mg/day, amitriptyline 25 mg/day and gabapentin 300 mg/day. After 4 months, the prednisone dose was tapered to 10 mg/day, which was maintained until completion of the MDT. Skin lesions had improved significantly after 6 months of treatment, and full resolution of erythroderma and neuritis was achieved when he completed the 12 months of MDT. No recurrence of erythroderma or RR occurred up to the last visit. The ulnar claw required surgical decompression; orthopedic shoes and physiotherapy sessions were used as adjuvant therapies, improving the neural sequelae.

**Fig. 3:**
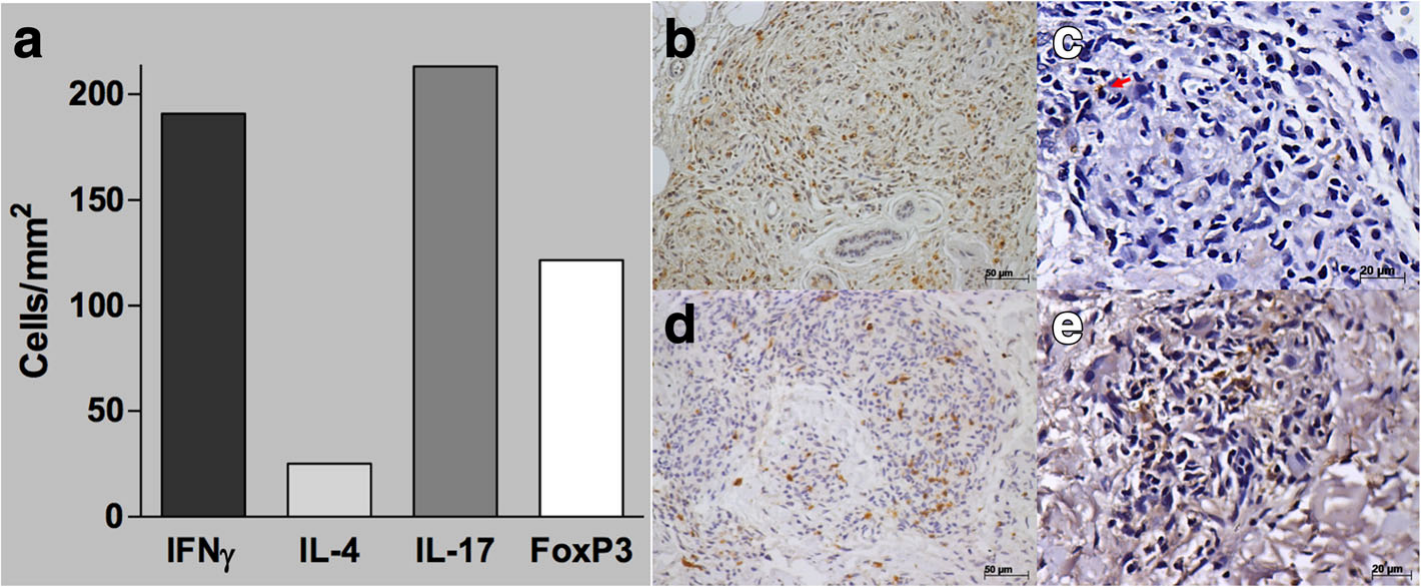
Immunohistochemistry findings. **a** In situ frequency of cytokines and FoxP3 expression (number of positive cells/mm^2^). Sections of immunohistochemistry staining for (**b**) anti-IFN-γ (Santa Cruz, Dallas, TX) (**c**) anti-IL-4 (Santa Cruz), (**d**) anti-IL-17 (R&D Systems, Minneapolis, MN) and (**e**) anti-FoxP3 (Ebioscience, San Diego, CA) monoclonal antibodies of a patient’s lesion biopsy. Slides were labeled with streptavidin–biotin complex (Dako, Carpinteria, CA). **b** and **d**, original magnification 200×; **a** and **c**, original magnification 400×. Numerous stained (brown) cells are found in (**b**) and (**d**), a more modest number is found in (**e**), while rare stained cells (arrow) were detected in (**c**)

Immunohistochemistry and peripheral blood cells cytometry were performed as previously described (Additional file 1) [5, 6]. During RR and erythroderma, IFNγ + lymphocytes and IL-17+ lymphocytes (191 and 213 cells/mm^2^, respectively) outnumbered IL-4+ lymphocytes and FoxP3+ lymphocytes (25 and 121 cells/mm^2^, respectively) in the skin tissue (Fig. 3). Our previous study of leprosy patients with severe RR (without erythroderma, RR group, *n* = 14) showed the opposite: FoxP3+ lymphocytes prevailed over IL-17+ lymphocytes (170 ± 23 vs. 114 ± 27 cells/mm^2^) [6]. Patient’s circulating Tregs were also reduced as compared with the RR group (2.17% vs. 3.4 ± 0.4%). However, the patient’s Tregs expanded normally upon in vitro stimulation with mitogen (phytohemagglutinin) or *M. leprae* antigens (data not shown).

The CARE guidelines were followed in this article.

## Discussion and conclusions

The immunological mechanisms underlying erythroderma and RR are not well established. RR would represent episodes of exacerbated Th-1 responses triggered by release of antigens from bacilli killed either by mycobactericidal drugs or by spontaneous flare-ups of the antimycobacteria immunoreactivity in patients with borderline (unstable) immunity [7, 8]. In erythroderma, also a state of immune dysregulation, this issue is complicated by the fact that these conditions are caused by many different diseases.

This case-report brings some unusual immunological findings that probably reflect the interplay between leprosy and erythroderma. Histopathology analysis revealed compact epithelioid granulomas, suggesting an efficient immune response, but also presence of isolated and grouped bacilli (viable and degenerated) within nerves, suggesting a yet active mycobacterial infection. This duality probably reflects the patient’s transition from an anergic/multibacillary state to a state of more effective immune response and bacillary control.

However, the simultaneous development of erythroderma indicates that this transition evolved through a dysregulated immune response. This is supported by the marked in situ over-expression of IFN-γ compared with IL-4 (IL-4/IFN-γ=0.13), consistent with the in situ pattern seen in RR in borderline leprosy, but not in erythroderma patients [9]. It has been reported that benign forms of erythroderma (eg, idiopathic or caused by atopic dermatitis) present slight predominance of IFN-γ over IL-4 expression (IL-4/ IFN-γ =0.6 and 0.9, respectively), while in malignant forms (eg, Sézary syndrome) IL-4 predominated (IL-4/ IFN-γ =1.8) [10]. However more recently, other authors, using Th-1 and Th-2-specific transcription factors, showed that in erythrodermic psoriasis and atopic dermatitis Th-2 responses predominated largely over Th-1 responses [11]. Although the data on erythroderma are still scarce and controversial, they markedly differ from those seen in our patient. These differences probably reflect the immune response pattern of the underlying disease.

Tregs and Th-17 responses appear to be reciprocally regulated in leprosy [6, 12, 13]. Another unexpected finding in this patient was the in situ over-expression of IL-17+ T-cells compared with Tregs, which contrasts with our previous findings showing that in borderline leprosy patients development of RR is associated with the concomitant decrease in the frequency of IL-17+ T-cells and the increase in the frequency of Tregs in the lesions [6]. In addition, the patient’s number of circulating Tregs was decreased when compared with our previous data on the RR group [6]. These alterations seem to be a direct effect of the patient’s immunological environment because the capacity of the patient’s Tregs to expand in vitro was preserved.

The balance among distinct T-cell subtypes that participate in a host’s cell-mediated immune response is tightly regulated through well-defined counter-regulatory mechanisms such as those that exist between Th-1 and Th-2 and between Th-17 and Tregs [14, 15]. We thus propose that the mechanisms underlying cutaneous immunological phenomena that culminate with erythroderma in our patient would be triggered by the flare-up of anti-mycobacteria Th-1 immune response. This blocked the local Th-2 immunity, favoring full expression of Th-17 responses while inhibiting the Treg pathway. Inhibition of the latter would, in turn, leave unchecked the cutaneous Th-1/Th-7 inflammation and subsequent development of erythroderma. We also hypothesize that MDT, concomitant with prednisone used for controlling the RR, contributed to the clinical improvement of erythroderma by reducing the mycobacteria burden. Further studies of case series of such patients are needed to confirm our hypothesis, since it was based on a single observation.

In conclusion, this case report highlights the importance of a thorough clinical examination of erythrodermic patients in search for its etiology and suggests that an intense and probably uncontrolled leprosy RR can culminate in the development of erythroderma.

## Additional file

Additional file 1: Immunohistochemistry staining for Foxp3, IL-4, IL-17 and IFN-γ of the biopsies of skin lesions. Material and methods for the immunohistochemistry stainings. (DOCX 16 kb)

## Abbreviations

MDT: Multidrug therapy
RR: Reversal reaction
Th: T-helper
