# Supplementary material for: A case report of erythroderma in a patient with borderline leprosy on reversal reaction: a result of the exacerbated reaction?
Source: BMC Dermatol. 2017 Dec 20;17:16. doi: 10.1186/s12895-017-0068-3 (PMC5738805; doi:10.1186/s12895-017-0068-3)
Supplement: Additional file 1: — Immunohistochemistry staining for Foxp3, IL-4, IL-17 and IFN-γ of the biopsies of skin lesions. Material and methods for the immunohistochemistry stainings. (DOCX 16 kb) [file 12895_2017_68_MOESM1_ESM.docx]

**Additional file 1**

**Immunohistochemistry staining for Foxp3, IL-4, IL-17 and INF-γ of the biopsies of skin lesions**

Immunohistochemistry staining out as previously described. (1, 2) Biopsies were taken with a standard dermatologic biopsy punch. Briefly, after deparafinization in xylene and hydration in ethyl alcohol, endogenous peroxydase was blocked by incubating in 3% hydrogen peroxydase solution in dark chamber. Antigen recovery was performed in a retrieval solution at pH 9.0 (S2368; Dako, Carpinteria, CA, USA) for 20 minutes at 95°C. Nonspecific proteins were blocked by incubating sections in skim milk. The primary antibody (anti-IL-4 [AF-204-NA, R&D Systems], anti-IFN-γ [ SC-8308, Santa Cruz Biotechnology], anti-IL-17 [AF-317-NA; R&D Systems] or anti-Foxp3 [14-4776; eBioscience] as well as a labeled streptavidin–biotin complex (LSAB; Dako) were then applied. 3,3-diaminobenzidine tetrahydroxychloride (DAB; Sigma) was used as chromogen, and the slides were counterstained with hematoxylin and hydrated in alcohol. The reaction was performed with positive and negative controls. The latter comprised an isotype control and omission of the primary antibody. The images were captured using AxioVision 4.8.2 software (Zeiss, Oberkochen, Germany). The area of the granulomatous inflammatory infiltrate was measured and the stained cells were counted using Image-Pro Plus, version 6.0 Media Cybernetics, Rockville, MD, USA.

References

# [Vieira AP](https://www.ncbi.nlm.nih.gov/pubmed/?term=Vieira%20AP%5BAuthor%5D&cauthor=true&cauthor_uid=26903606), [Trindade MA](https://www.ncbi.nlm.nih.gov/pubmed/?term=Trindade%20M%C3%82%5BAuthor%5D&cauthor=true&cauthor_uid=26903606), [Pagliari C](https://www.ncbi.nlm.nih.gov/pubmed/?term=Pagliari%20C%5BAuthor%5D&cauthor=true&cauthor_uid=26903606), [Avancini J](https://www.ncbi.nlm.nih.gov/pubmed/?term=Avancini%20J%5BAuthor%5D&cauthor=true&cauthor_uid=26903606), [Sakai-Valente NY](https://www.ncbi.nlm.nih.gov/pubmed/?term=Sakai-Valente%20NY%5BAuthor%5D&cauthor=true&cauthor_uid=26903606), [Duarte AJ](https://www.ncbi.nlm.nih.gov/pubmed/?term=Duarte%20AJ%5BAuthor%5D&cauthor=true&cauthor_uid=26903606), [Benard G](https://www.ncbi.nlm.nih.gov/pubmed/?term=Benard%20G%5BAuthor%5D&cauthor=true&cauthor_uid=26903606). Development of Type 2, But Not Type 1, Leprosy Reactions is Associated with a Severe Reduction of Circulating and In situ Regulatory T-Cells. [Am J Trop Med Hyg.](https://www.ncbi.nlm.nih.gov/pubmed/26903606) 2016; 94(4):721-7.

1. Palermo ML, Pagliari C, Trindade MA, Yamashitafuji TM, Duarte AJS, Cacere CR, Benard G. [Increased Expression of Regulatory T Cells and Down-Regulatory Molecules in Lepromatous Leprosy](https://www.ncbi.nlm.nih.gov/pmc/articles/PMC3335697/). Am J Trop Med Hyg. 2012; 86(5): 878–883.
